# A Two-Stage MCDM Model for Exploring the Influential Relationships of Sustainable Sports Tourism Criteria in Taichung City

**DOI:** 10.3390/ijerph17072319

**Published:** 2020-03-30

**Authors:** Jen-Jen Yang, Yen-Ching Chuang, Huai-Wei Lo, Ting-I Lee

**Affiliations:** 1 Office of Physical Education General Education Center, Chaoyang University of Technology, 168, Jifeng E. Rd., Wufeng District, Taichung 413310, Taiwan; jjyang@cyut.edu.tw; 2 Graduate Institute of Urban Planning, College of Public Affairs, National Taipei University, 151, University Rd., Sanxia District, New Taipei City 23741, Taiwan; yenching.chuang@gmail.com; 3 Department of Industrial Engineering and Management, National Taipei University of Technology, 1, Sec. 3, Zhongxiao E. Rd., Taipei 10608, Taiwan; w110168888@gmail.com

**Keywords:** sustainable sports tourism, sports for all, MCDM, Bayesian BWM, rough DEMATEL

## Abstract

Many countries advocate sports for all to cultivate people’s interest in sports. In cities, cross-industry alliances between sports and tourism are one of the common practices. The following two important issues need to be discussed, namely, what factors should be paid attention to in the development of sports tourism, and what are the mutual influential relationships among these factors. This study proposes a novel two-stage multi-criteria decision-making (MCDM) model to incorporate the concept of sustainable development into sports tourism. First, the Bayesian best–worst method (Bayesian BWM) is used to screen out important criteria. Bayesian BWM solves the problem of expert opinion integration of conventional BWM. It is based on the statistical probability to estimate the optimal group criteria weights. Secondly, the rough decision making trial and evaluation laboratory (rough DEMATEL) technique is used to map out complex influential relationships. The introduction of DEMATEL from the rough set theory has better practicality. In the calculation program, interval types are used to replace crisp values in order to retain more expert information. A city in central Taiwan was used to demonstrate the effectiveness of the model. The results show that the quality of urban security, government marketing, business sponsorship and mass transit planning are the most important criteria. In addition, in conjunction with local festivals is the most influential factor for the overall evaluation system.

## 1. Introduction

Including some sports activities or watching sports events in the tourist itinerary has become one of the major development projects of the tourism industry [1]. Sports tourism is defined as “combining sports events with tourism” and can be divided into six types, including sports events, sports resorts, sports cruises, sports attractions, sports adventures and sports tours [2]. Many studies have pointed out that organizing related sports tourism in cities is conducive to the development of social image and local economy. For this reason, many cities have also set up special organizations to organize sports events to increase regional exposure and sports image [3,4]. Many countries are actively seeking viable marketing strategies to attract foreign and domestic tourists to travel there. The most typical way is to increase the number of tourists by using sports events. For example, in 2017, Taipei hosted a 13-Erlenmeyer-day Universiade and sold a total of 720,000 tickets to the sports event. This event not only attracted more foreign tourists but also promoted the local culture of Taipei. In addition, some well-known cities have been successfully transformed into sports tourism attractions and have established their image as sports cities. For example, Perth is known as the City of Sporting Events, Lausanne is known as Olympic Capital City and Lake Placid is billed as the Winter Sports Capital of the United States [2].

Due to abnormal climate change and frequent natural disasters, many international organizations (such as the World Health Organization (WHO), European Union (EU) and World Trade Organization (WTO)) have called on all industries to pay attention to “sustainable development” and formulated many regulations and agreements on environmental protection [5]. Therefore, the tourism industry is also actively moving towards the developmental vision of sustainable tourism and many kinds of research on sustainable sports tourism have been released. Gibson et al. [6] explored the cooperation between six small-scale sports events and local sports agencies (the evaluation includes economic, social and environmental protection aspects) and surveyed 447 sports event participants and spectators in terms of sports planning satisfaction. Pouder et al. [3] used expert interviews to explore how for-profit organizations can develop the market for sports tourism. Their study is particularly focused on economic development, with the goal of maximizing returns. Gil-Alana et al. [4] examined whether fluctuations in financial exchange rates have a significant impact on the returns of Brazilian sports tourism. The study used a multiple linear regression model to analyze the structure of tourism revenue structure over 20 years. Hsu et al. [7] developed an island-type sustainable tourism attitude scale focusing on the environmental protection perspective of sports attractions. Their data came from a survey of three islands in Taiwan. The results show that local culture and environmental protection are the most important factors in tourism development.

It is an important task to develop an effective urban tourism development evaluation model [8,9]. Multi-criteria decision-making (MCDM) is widely used in various evaluation and selection problems and it has excellent evaluation performance under many constraints. In contrast to statistical methods, MCDM does not need to establish basic assumptions for criteria or variables. MCDM has developed many soft calculation methods to process a variety of complex data (including data from expert interviews and data from actual surveys) and provide valuable management information to support decision-makers in formulating optimal strategies [10,11,12,13]. At present, there have been some studies using MCDM to study tourism-related issues, such as surveys of service quality in tourism [14], hotel performance evaluation and selection [15], tourism development and management [16].

According to the literature review, the evaluation system of urban sustainable sports tourism for cities has not been established yet. The purpose of this study is to propose a novel two-stage MCDM model to establish the evaluation criteria for cities to develop sustainable sports tourism and to explore the mutual influential relationships among the criteria. The evaluation framework is new, and the proposed hybrid methodology has not appeared. In summary, this model brings some contributions and innovations to sustainable tourism development for the cities:

(i) The traditional sustainability concept revolves around social (S), environmental (B) and economic (C). The addition of “institutional sustainability (I)” makes the evaluation structure more complete;
(ii) Bayesian best–worst method (Bayesian BWM) [17] is used as a criteria screening method. Compared to the analytic hierarchy process (AHP), the number of pairwise comparisons questionnaire content in the Bayesian BWM is significantly reduced and it has better consistent results;
(iii) In the calculation of DEMATEL, it combines rough set theory to optimize the applicability of the conventional DEMATEL;
(iv) The mutual influential relationships among the criteria are identified using rough DEMATEL to support decision-makers in developing urban sports tourism development strategies;
(v) The proposed methodology is not limited to any industry and various industries can imitate and develop their own decision-making systems.

The rest of this article is organized as follows. Section 2 briefly reviews the literature on sports tourism and presents the proposed evaluation framework for sustainable urban sports tourism development. Section 3 introduces the proposed two-stage MCDM model, including the implementation steps of Bayesian BWM and rough DEMATEL. Section 4 presents a real case in Taiwan to illustrate the feasibility and practicality of the proposed model. Section 5 includes discussions and management implications. Section 6 presents concludes with conclusions and future research.

## 2. Literature Review for Sustainable Sports Tourism Evaluation Criteria

Many countries promote sports tourism by joining sports, setting up specialized sports tourism agencies whether in large cities or local towns [3,18]. Sports tourism is a special type of tourism that provides tourists with an active (active participation in sports events as competitors) or passive (passive participation in sports events as spectators) experience that is different from traditional tourism. People interact with events, people and places when participating in sports tourism-related events [19]. Kim et al. [20] pointed out that large-scale sports tourism activities can attract many domestic and foreign participants and spectators; these sports events can increase local income and opportunities for urban development. On the contrary, these events also have negative impacts, that is, traffic congestion, environmental pollution, safety issues and damage to residents’ rights. Therefore, the concept of sustainable development combined with research on tourism has been proposed. Nunkoo et al. [21] emphasized that the establishment of public trust and the formulation of environmental protection policies can develop excellent urban tourism. Gkoumas [22] proposes a comprehensive assessment index for sustainable tourism for the Mediterranean tourism industry; local governance is the most critical factor for the development of sports tourism. Musavengane et al. [23] explored the security of tourism in African countries, holding that cultural tolerance, local security, medical and rescue flexibility and the integrity of environmental awareness are all key items for evaluation. Yang et al. [24] first proposed a complete MCDM model of sustainable sports tourism, which established an effective evaluation system for tourist attractions in central Taiwan. Unfortunately, to our knowledge, no article has been conducted to examine the performance of sustainable sports tourism in the cities. In addition, the mutual influential relationships among evaluation criteria have not been explored.

This study proposes a novel evaluation framework to determine the evaluation criteria and their mutual influential relationships. For cities to develop sustainable sports tourism, they must receive support from local governments and the tourism industry. First, important criteria should be fully integrated into the evaluation system to reflect the characteristics and connotation of sports tourism. The initial criteria review was based on relevant academic literature [3,8,9,18,19,24] and expert interviews (a decision group was formed, including tourism industry, Taiwan Tourism Bureau, local government and environmental protection experts). The main framework includes four dimensions, namely social (S), environmental (G), economic (E) and institutional (I). Each of these four dimensions contains several criteria and a total of 30 evaluation criteria are included in the evaluation framework. The criteria, descriptions and literature are shown in Table 1.

## 3. The Proposed Two-Stage MCDM Model

The chosen case is Taichung City, Taiwan. The Taichung City Government actively promotes sports infrastructure and promotes the correct sports concept to implement “sports for all ages”. In December 2019, the Taichung City Sports Bureau decided to organize marathons to connect the sports events with local specialties in order to serve the purpose of marketing the city and promoting culture. In 2020 alone, Taichung City has already prepared at least 35 marathon events. However, building an image of a sports city is a difficult and complex project; many factors and restrictions must be considered, including economic feasibility, social development, environmental awareness and policy support. Only through continuous review and improvement can we move towards the vision of urban sports for all ages. At present, there has not been a sustainable sports tourism evaluation system developed specifically for the cities. In addition, most studies have not examined the mutual influential relationships among criteria. Which evaluation criteria are the main factors that affect the success or failure of urban sports tourism? How do these criteria affect other criteria? These two issues are the focal points of this study.

In the study, the decision-making team consisted of ten experts, including tourism managers, members of the Ministry of Tourism and academics. These ten experts have at least 8 years of qualifications in sports events or tourism-related jobs; their current jobs are highly relevant to the development of sports tourism. The proposed evaluation framework is presented in Section 2 and Section 4 dimensions with 30 criteria classified under them were identified.

**Figure 1:**
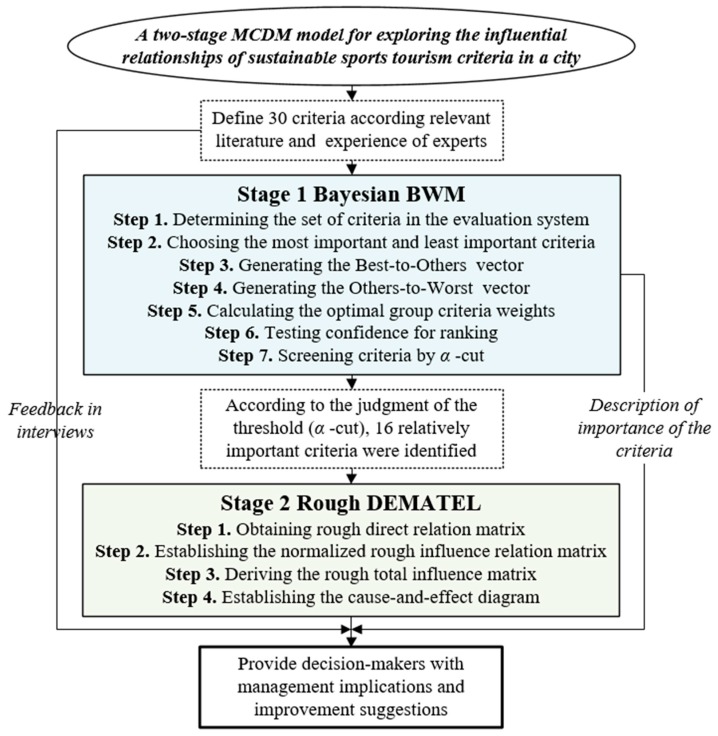
The analysis procedure diagram.

Next, we describes the method used and its detailed calculation process. In the first stage, preliminary evaluation criteria were established based on the literature discussions on sports tourism and sustainable tourism. Due to the large number of evaluation criteria, screening must be performed to retain key criteria. Based on the interview data of several experts, the Bayesian BWM was used to calculate the weight of criteria and select the key criteria. Bayesian BWM—proposed by Mohammadi and Rezaei [17]—effectively integrates the judgments of multiple experts and shortens the computational procedures of the conventional BWM. In the second stage, the rough decision making trial and evaluation laboratory (rough DEMATEL) technique is used to map a cause-and-effect diagram of criteria to examine the strength of the influential relationship among the criteria. This study combines rough set theory with conventional DEMATEL. On the one hand, the consensus of the decision-making group can be known. Moreover, the interval value operation can be retained to avoid the loss of information. The analysis procedure diagram of this study is shown in Figure 1.

### 3.1. Stage 1: Bayesian BWM

Bayesian BWM effectively integrates the opinions of multiple experts to generate a set of optimal group criteria weights. Its survey process is simple and intuitive. Experts are asked to choose the most important and least important criteria; then they are compared pairwise with other criteria to form a structured set of two vectors. Based on the concept of statistical distribution, the optimal group criteria weights are estimated. The detailed Bayesian BWM derivation and proof can be found in the study of Mohammadi and Rezaei [17]. The implementation steps of Bayesian BWM are explained as follows:

#### 3.1.1. Step 1. Determining the Set of Criteria in the Evaluation System

The evaluation criteria $\left\{c_{1},\;c_{2},\ldots ,\;c_{n}\right\}$ were identified through literature review and multiple expert interviews.

#### 3.1.2. Step 2. Choosing the Most Important and Least Important Criteria

Based on the set of criteria, each expert chooses what s/he considers the most important and least important criteria.

#### 3.1.3. Step 3. Comparing the Most Important Criteria with Other Criteria to Generate the BO (Best-to-Others) Vector

Each expert evaluates the relative importance of the most important criteria to other criteria to generate the BO vector. The ratings of BWM are shown in Table 2.

(1) $$A_{Bj}=\left(a_{B1},\;a_{B2},\ldots ,\;a_{Bn}\right)$$

where $a_{Bj}$ indicates the importance of the most important criterion *B* relative to criterion *j*.

#### 3.1.4. Step 4. Comparing Other Criteria with the Least Important Criteria to Generate the OW (Others-to-Worst) Vector

Similar to Step 3, each expert evaluates the relative importance of the other criteria to the least important criteria to generate the OW vector.

(2) $$A_{jW}={\left(a_{1W},\;a_{2W},\ldots ,\;a_{nW}\right)}^{T}$$

where $a_{jW}$ indicates the importance of the other criterion *j* relative to the least important criterion *W*.

#### 3.1.5. Step 5. Calculating the Optimal Group Criteria Weights

Each expert follows Step 1 to Step 4 to get multiple sets of BO and OW vectors. According to the MATLAB program software provided by Mohammadi and Rezaei [17], the evaluation scores of all experts are used as input data to obtain the optimal group criteria weights.

#### 3.1.6. Step 6. Testing Confidence for Ranking

After the weights are obtained, it must be checked whether the ranking of the weight is consistent. Assume that the two criteria in the criteria set are *c_i_* and *c_j_* and use the concept of Credal Ranking to test their confidence. Then the probability that *c_i_* is better than *c_j_* is

(3) $$P\left(c_{i}>c_{j}\right)=\int I\left(w_{i}^{agg}>w_{j}^{agg}\right)P\left(w^{agg}\right)$$

where $w^{agg}$ is the group criteria weight, $P\left(w^{agg}\right)$ is the posterior probability of $w^{agg}$ and *I* is the condition parameter, which can be calculated when $\left(w_{i}^{agg}>w_{j}^{agg}\right)$ is true, otherwise it is 0. The Markov-chain Monte Carlo (MCMC) technique is used to perform multiple simulations and the number of samples *Q* obtained by it is used to calculate its average confidence level.

(4) $$P\left(c_{i}>c_{j}\right)=\frac{1}{Q}\sum_{q=1}^{Q}I\left(w_{i}^{agg_{q}}>w_{j}^{agg_{q}}\right);\;P\left(c_{j}>c_{i}\right)=\frac{1}{Q}\sum_{q=1}^{Q}I\left(w_{j}^{agg_{q}}>w_{i}^{agg_{q}}\right)$$

where $w^{agg_{q}}$ represents *q* $w^{agg}$ s from the MCMC sample. When $P\left(c_{i}>c_{j}\right)>0.5$, it indicates that criterion *i* is more important than criterion *j* and the probability presented is the confidence level. In addition, the total probability is 1, $P\left(c_{i}>c_{j}\right)+P\left(c_{j}>c_{i}\right)=1$.

#### 3.1.7. Step 7. Screening Criteria by α-cut

The α-cut is the threshold value of the screening criteria. There are *n* criteria in the criteria set, $\left\{c_{1},\;c_{2},\ldots ,\;c_{n}\right\}$. α-cut is shown below.

(5) $$\alpha \text{-}\mathrm{cut}=\frac{1}{n}$$

This step can distinguish the relatively important and relatively unimportant criteria groups. We retain the rules that are larger than α-cut.

### 3.2. Stage 2: Rough DEMATEL

DEMATEL technique was proposed by Battelle Memorial Institute in 1972. This method is used to solve the problem of the complex structures in real society [25]. DEMATEL aims to establish a structure diagram that can show mutual influential relationships among the criteria. It is called a cause-and-effect diagram, which effectively supports decision-makers in understanding the interaction and influence relationships in the entire system. The conventional DEMATEL uses arithmetic average method to integrate evaluation data from multiple experts. This study combines rough set theory with DEMATEL, called rough DEMATEL or R-DEMATEL. This method not only can know the consensus degree of the decision-making group, but also retain the calculation of interval values to avoid the loss of information. The calculation steps of the rough number can be found in Lo et al. [26] and Chang et al. [27]. We use a simple example to illustrate how to integrate the rough number calculations of multiple experts. Assume that the evaluation values of the five experts in evaluating event *A* are 4, 4, 3, 2 and 2, respectively, then lower and upper bounds of rough numbers ($\underline{Lim}$ and $\bar{Lim}$) are

$$\begin{array}{l} \underline{Lim}\left(4\right)=\left(4+4+3+2+2\right)/5=3,\;\bar{Lim}\left(4\right)=\left(4+4\right)/2=4\\ \;\Rightarrow {\tilde{A}}^{\left(1\right)}={\tilde{A}}^{\left(2\right)}=\tilde{4}=\left[3,\;4\right]; \end{array}$$

$$\begin{array}{l} \underline{Lim}\left(3\right)=\left(3+2+2\right)/3=2.333,\;\bar{Lim}\left(3\right)=\left(3+4+4\right)/3=3.667\\ \;\Rightarrow {\tilde{A}}^{\left(3\right)}=\tilde{3}=\left[2.333,\;3.667\right]; \end{array}$$

$$\begin{array}{l} \underline{Lim}\left(2\right)=\left(2+2\right)/2=2,\;\bar{Lim}\left(2\right)=\left(4+4+3+2+2\right)/5=3\\ \;\Rightarrow {\tilde{A}}^{\left(4\right)}={\tilde{A}}^{\left(5\right)}=\tilde{2}=\left[2,\;3\right]. \end{array}$$

where the symbol “~” indicates that those are rough numbers. This set of scores can be obtained by averaging as follows:

$$\tilde{A}=\left[\left(3+3+2.333+2+2\right)/5,\;\left(4+4+3.667+3+3\right)/5\right]=\left[2.467,\;3.533\right].$$

After screening criteria by Bayesian BWM, the rough DEMATEL procedure is further performed. The detailed steps are stated below:

#### 3.2.1. Step 1. Obtaining Rough Direct Relation Matrix $\tilde{Q}$

After screening, there are $n^{*}$ criteria and each expert evaluates the direct impact of the criteria i on the criteria j according to DEMATEL’s evaluation ratings (Table 3). Then, the subjective opinions of all experts will be converted into a set of interval-type interval numbers by the rough number operation in rough theory and a rough direct relation matrix $\tilde{Q}$ can be obtained. As shown in Equation (6).

(6) $$\tilde{Q}={\left[{\tilde{q}}_{ij}\right]}_{n^{*}\times n^{*}},i=j=1,2,\ldots ,n^{*}$$

where ${\tilde{q}}_{ij}=[q_{ij}^{L},q_{ij}^{U}]$.

#### 3.2.2. Step 2. Establishing the Normalized Rough Influence Relation Matrix $\tilde{D}$

The rough direct relation matrix $\tilde{Q}$ can obtain a normalized rough influence relation matrix $\tilde{D}$ through Equations (7) and (8). The normalized program can convert the evaluation value to between 0 and 1.

(7) $$\tilde{D}=\varepsilon \times \tilde{Q}$$

(8) $$\varepsilon =\min\;\left\{1/\underset{i}{\max}\sum_{j=1}^{n^{*}}q_{ij}^{U},1/\underset{j}{\max}\sum_{i=1}^{n^{*}}q_{ij}^{U}\right\},i=j=1,2,\ldots ,n^{*}$$

where $\tilde{D}={\left[{\tilde{d}}_{ij}\right]}_{n^{*}\times n^{*}},\;0\leq {\tilde{d}}_{ij}<1$ and ${\tilde{d}}_{ij}=\left[d_{ij}^{L},d_{ij}^{U}\right]$. In $\sum_{j=1}^{n^{*}}d_{ij}^{U}$ and $\sum_{i=1}^{n^{*}}d_{ij}^{U}$, the sum of any row or column is less than or equal to 1.

#### 3.2.3. Step 3. Deriving the Rough Total Influence Matrix $\tilde{T}$

The normalized rough influence relation matrix $\tilde{D}$ uses Equation (9) to calculate the degree of each direct influence relationship and indirect influence relationship (I is the identity matrix) and finally integrates a rough total influence matrix $\tilde{T}$ as shown in Equation (10).

(9) $$\begin{array}{ll} \tilde{T} & =\tilde{D}+{\tilde{D}}^{2}+\cdots +{\tilde{D}}^{\Theta }=\tilde{D}\left(I+\tilde{D}+{\tilde{D}}^{2}+\cdots +{\tilde{D}}^{\Theta -1}\right)\\ & =\tilde{D}\left(I-{\tilde{D}}^{\Theta }\right){\left(I-\tilde{D}\right)}^{-1}=\tilde{D}{\left(I-\tilde{D}\right)}^{-1},\mathrm{when}\;\Theta \to \infty ,{\tilde{D}}^{\Theta }={\left[0\right]}_{n^{*}\times n^{*}} \end{array}$$

(10) $$\tilde{T}={\left[{\tilde{t}}_{ij}\right]}_{n^{*}\times n^{*}}$$

where ${\tilde{t}}_{ij}=\left[t_{ij}^{L},t_{ij}^{U}\right]$.

#### 3.2.4. Step 4. Establishing the Cause-and-Effect Diagram

The rough total influence matrix $\tilde{T}$ can obtain the degree of rough affecting relationship (${\tilde{s}}_{i}$) and the degree of rough affected relationship (${\tilde{o}}_{i}$) of each criterion through Equations (11) and (12).

(11) $$\tilde{s}={\left[{\tilde{s}}_{i}\right]}_{n^{*}\times 1}$$

(12) $$\tilde{o}={\left[{\tilde{o}}_{j}\right]}_{1\times n^{*}}^{T}={\left[{\tilde{o}}_{i}\right]}_{n^{*}\times 1}^{}$$

where the symbol “*T*” stands for transpose. In addition, ${\tilde{s}}_{i}=\left[\sum_{j=1}^{n}t_{ij}^{L},\sum_{j=1}^{n}t_{ij}^{U}\right]$ and ${\tilde{o}}_{j}={\left[\sum_{i=1}^{n}t_{ij}^{L},\sum_{i=1}^{n}t_{ij}^{U}\right]}^{T}$.

The ${\tilde{s}}_{i}+{\tilde{o}}_{i}$ represents the rough total influence of the criterion within the evaluation system, and is called the prominence. ${\tilde{s}}_{i}-{\tilde{o}}_{i}$ represents the rough net influence of the criterion within the evaluation system and is called the net cause-effect. If ${\tilde{s}}_{i}-{\tilde{o}}_{i}>0$, it represents the degree of rough net influence of the criterion on other criteria; on the contrary, if ${\tilde{s}}_{i}-{\tilde{o}}_{i}<0$ it represents the degree of rough net influence of the criterion by other criteria. The detailed cause-and-effect diagram results are presented in Section 4.2.

## 4. Empirical Example

Participating in sports activities not only can promote the physical health of people of all ages, but also bring social benefits and improve people’s happiness. Healthy people will be the biggest asset of a country; the physical fitness of the people will be the foundation of the country’s competitiveness. Moderate exercise promotes physiological metabolism and helps to resist stress. In order to enhance the country’s sports competitiveness and protect people’s sports rights, the promotion of sports has become the focal policy of advanced countries to learn and observe from each other. In Taiwan, the most common sports activities include outdoor leisure sports, ball sports, stretching, dancing, water sports and so on. Among them, outdoor leisure sports account for more than 80% of total sports events [28]. Therefore, the sports projects that this study explores to promote sustainable sports tourism are mainly outdoor leisure sports. This section introduces the background of the case, as well as the practical application of Bayesian BWM and rough DEMATEL.

### 4.1. Screening the Criteria by Using Bayesian BWM

Based on the Bayesian BWM calculation described in Section 3.1, first, each expert was required to make pairwise comparisons of the criteria in each dimension. A total of four BWM questionnaires needed to be filled out. Since the function of Bayesian BWM at this stage is for screening criterion, there is no need to perform pairwise comparisons for the dimensions. Consistency ratio (CR) was performed on the recovered BWM questionnaires to check the logic of the experts in the response process. Based on the consistency test formula proposed by Rezaei [29], the average CR value in the study is 0.014 (with high consistency). Table 4 lists the optimal group criteria weights. According to the judgment of the threshold (α-cut), 16 relatively important criteria were identified, which are important factors for the sustainable development of urban tourism, including S6, S7, S8, G1, G2, G4, G6, G8, E4, E5, E6, E7, I1, I2, I4 and I7. The mutual influential relationships of these criteria included in the evaluation system were analyzed by the rough DEMATEL technique.

In order to check whether the weights obtained, and the ranking are reliable, a ranking confidence test is performed. Among the four dimensions, their confidence levels of ranking are 0.926, 0.871, 0.868 and 0.904, respectively. It represents the criteria ranking in each dimension is highly confident. Next, rough DEMATEL analysis was performed on the criteria incorporated in the evaluation system.

This study also compared the criteria screening results of AHP, conventional BWM and Bayesian BWM, as shown in Table 5.

AHP and BWM have fewer screening criteria than Bayesian BWM (without G8 and I2). This is because AHP and BWM use arithmetic averages when integrating experts’ opinions. This method is vulnerable to the influence of extreme values, resulting in the loss of some information. In contrast, Bayesian BWM, which pays extra consideration for G8 and I2, makes the influential relationship system of the criteria more complete. It must be noted here that I2 and G8 are important affecting and affected factors in the analysis of rough DEMATEL.

### 4.2. Obtaining the Cause-and-Effect Diagram by Using Rough DEMATEL

The implementation process of rough DEMATEL is explained in Section 3.2. The data of 10 experts’ surveys are calculated according to this process to obtain the rough influence degree of each criterion, as shown in Table 6.

The consensus degree of the experts can be viewed by average sample gap index (${\left(n\left(n-1\right)\right)}^{-1}\times \sum_{i=1}^{n}\sum_{j=1}^{n}\left(\left|t_{ij}^{p}-t_{ij}^{p-1}\right|/t_{ij}^{p}\right)\times 100\%$), where *n* is the number of samples, *p* is the number of experts and *t* is the evaluation value in the matrix. Based on this index, the average gap of the 10 experts is 4.8%, which means the confidence level is 95.2%, indicating that these experts have a high degree of consensus.

**Figure 2:**
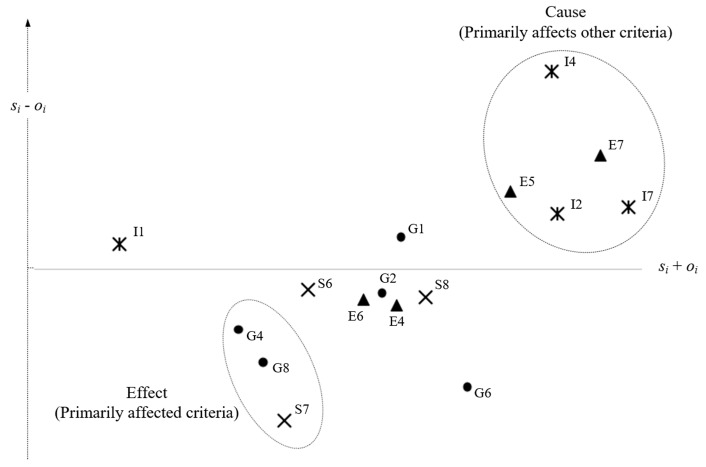
Cause-and-effect diagram of criteria.

Table 6 shows the total influence (${\tilde{s}}_{i}+{\tilde{o}}_{i}$) and net influence (${\tilde{s}}_{i}-{\tilde{o}}_{i}$) for all criteria. The larger ${\tilde{s}}_{i}-{\tilde{o}}_{i}$, the greater the degree to which this criterion affects other criteria. In addition, ${\tilde{s}}_{i}+{\tilde{o}}_{i}$ can indicate the total influence in the overall evaluation system to show the proportion of importance. We use ${\tilde{s}}_{i}+{\tilde{o}}_{i}$ as the horizontal axis and ${\tilde{s}}_{i}-{\tilde{o}}_{i}$ as the vertical axis to draw the cause-and-effect diagram of the criteria, as shown in Figure 2.

This approach allows policy-makers to quickly understand which criteria are the main causes and which are the effects to support the formulation of an appropriate management strategy. In Figure 2, the upper-right criteria indicate a high total influence and net influence, which are the main causes. In contrast, the lower-left criteria indicate lower total and net influences, which are the effects. Obviously, I4 is the most important affecting factor for cities to promote sustainable sports tourism and the rest are E7, I7, E5 and I2. In addition, G4, G8, S7 are the factors most affected by other criteria. The management implications derived from rough DEMATEL’s analysis are discussed in Section 5.

## 5. Discussion

Sports will positively change a person’s physical fitness and mental state [30,31]. The awakening of the consciousness of “sports for all” has forced major cities to invest resources to host sports events and thus shape the image of the sports cities. In order to achieve sustainable urban development, economic, social and environmental aspects are the main evaluation dimensions [8,9,24,32]. Many publications from the literature advocate the importance of institutional substantiality, so this study includes the institutional aspect as one of the evaluation dimensions to make the evaluation structure more comprehensive. By reviewing the literature and integrating the opinions of multiple experts, an evaluation system for sustainable urban tourism development was established. However, it is important to understand those criteria and to explore their mutual influential relationships. To our knowledge, these issues have not been studied and discussed.

This study proposes a two-stage MCDM decision model. Bayesian BWM is used to determine the importance weights of the criteria and rough DEMTAEL is used to identify the mutual influential relationships of the important criteria. The studies of Mohammadi and Rezaei [17] and Yang et al. [24] point out that Bayesian BWM solves the problem of integrating expert opinions for the conventional BWM and obtains a set of optimal group criteria weights. This study reduced 30 evaluation criteria to 16, which are relatively important criteria for measuring the performance of sustainable sports tourism. In terms of the Social (S) dimension, maintaining the quality of urban public order (S8) is the most important criterion in the evaluation system, with a weight of 0.223. This result echoes the findings of Gkoumas [22] and Musavengane et al. [23], where they mentioned that public order in the region affects the safety of the tourists. Some famous tourist attractions have had negative incidents, including theft, robbery, scams, traffic accidents, viral infections and racial discrimination. Before large-scale sports events are held, public security management must be strengthened and rigorous planning and control of personnel entry and exit to ensure passenger confidence in safety. In terms of the institutional (I) dimension, the development efficiency of sports tourism depends on the marketing and promotion by local governments (I7). In order to prevent urban tourism from falling into the off-season, periodic events should be organized to maintain the stability of the number of tourists. Sponsorship and support from local businesses (E5) is the most important criterion in the Economic (E) dimension. It is not difficult to understand that business sponsorship often brings more and more resources to sports activities. The sponsors and the organizers can achieve a win-win result by mutual benefit; for the participants, they can further understand the sponsor brands and experience their products. When it comes to environmental protection (G), planning for the city’s mass transit system (G6) helps reduce the city’s transportation carbon emissions and noise. At present, many environmental sports events have promoted zero-pollution itineraries. The measures include using electric vehicles, not using plastic materials and using recyclable containers.

Rough DEMATEL maps out the main causes and effects. The promotion of sustainable sports tourism in the cities must particularly focus on the following criteria: In conjunction with festivals in the city (I4), increasing the number of visits to the attractions in the city (E7), marketing and promotion by local governments (I7), sponsorship and support by local businesses (E5) and maintenance of the urban tourism website (I2). These criteria will affect the performance of other criteria. This result echoes the management implications of many studies, including Pouder et al. [3], Huang et al. [18], Lee and Xue [9] and Yang et al. [24]. The government must pay special attention to the performance of these five criteria. In order to allow the public to understand that the city is promoting sports tourism plans, print and online media promotion should be strengthened; sports events should be organized in conjunction with festivals. Business sponsorship also helps to increase the spread of sports ethos and makes the implementation of sports tourism plans more effective. In addition, for restrictions on plastic materials (G4), monitoring the quality of drinking water (G8) and formulating procedures for handling emergencies (S7), they require the development of other criteria to achieve high performance. The development of sports tourism in the city is a complex and difficult project: continuous simulation and review are required to make subsequent sports events more successful.

## 6. Conclusions

In summary, the two-stage evaluation model proposed in this study provides a complete and systematic method, providing the management implications of the development of sports tourism in the cities. This effective soft calculation method can reduce the subjectivity of management decisions. The academia has not yet studied and explored the mutual influential relationships among the criteria for sustainable sports tourism. Our model integrates several state-of-the-art methods and takes into account a variety of realistic factors, including the consideration of message uncertainty and the introduction of the concept of rough set theory.

It is well known that sports bring many benefits to people’s physiology and psychology. The spirit of sustainability has brought into the sports tourism industry the purpose of accelerating the expansion of the “sports for all”. This study proves the effectiveness and reliability of the proposed model. It should bring several benefits to practitioners and sports-related sectors: (i) identifying the most important and influential criteria; (ii) providing an improved basis for urban development sports tourism; (iii) helping decision-makers in the decision-making process to be more systematic.

In the future, researchers can further investigate the quantitative data of the actual assessment, making the evaluation results more accurate. Beyond that, using Bayesian BWM for cross-dimensional criteria comparison can entail more discussion.

## Figures and Tables

**Table 1:** Evaluation criteria and descriptions.

Dimension	Criteria	Description	References
Social (S)	Strengthening the image of the city (S1)	The culture of the region will affect the development of sports; it is necessary to strengthen the image of the city.	[9,18,19]
	Maintaining the lifestyle of urban residents (S2)	While promoting urban sports tourism, it is necessary to ensure that it does not affect the original lifestyle and quality of residents.	[18,19,22,24]
	Providing additional benefits for urban area residents (S3)	Providing additional benefits or subsidy programs for local residents, so that residents can better accept sports events and provide assistance.	[18,19,24]
	Promoting social equity (S4)	Respecting for equality and protection of participation rights of disadvantaged ethnic groups.	[18,19,24]
	Insuring for participants (S5)	Insuring for each participant and staff.	[18]
	Actively donating part of the income to public welfare (S6)	Some of the incomes from sports events will be donated to social welfare or public welfare organizations.	[18,19]
	Formulating procedures for handling emergencies (S7)	Prior to the event, all emergency situations must be prepared; handling procedures must be carefully planned.	[18,19,24]
	Maintaining the quality of urban public order (S8)	Paying attention to the law and order of the city to ensure that all event personnel can feel safe and secure.	[18,22,24]
Environmental (G)	Using the city’s existing infrastructure (G1)	New facilities or buildings should not be built for sporting events. The existing facilities should be used to maintain the original look of the city.	[3,9,18,19,24]
	Compliance with environmental protection regulations (G2)	All activities must be prepared in an environmentally friendly manner and must be as natural as possible.	[8,9,18,19,24]
	Developing protection measures for natural ecological areas (G3)	Establishing protection regulations for the city’s natural ecological area to ensure that the area is not damaged by activities.	[8,9,18,24]
	Restrictions on plastic materials (G4)	Consumables and items used in the event shall be controlled according to the amount of consumed plastic materials.	[9,18,19,24]
	Well-planned urban cleanup plan (G5)	Sports events bring crowds and waste; a complete cleaning plan should be developed to maintain the cleanliness of the city.	[8,9,18,24]
	Planning the city’s mass transit system (G6)	A sound mass transit system can effectively reduce the problem of traffic congestion and reduce carbon emissions from self-driving cars.	[8,9,19]
	Controlling noise pollution (G7)	Gathering of people will generate huge noise; noise control should be done at specific times and places.	[9]
	Monitoring the quality of drinking water (G8)	The source of drinking water and the filtration system should be controlled in detail to ensure the water quality of the participants.	[8]
Economic (E)	Providing information on accommodation in the city (E1)	Providing complete accommodation and related information to facilitate participants in planning their accommodation.	[18,19]
	Providing information on dining in the city (E1)	Providing comprehensive dining information and presenting local food and beverage to tourists from other places.	[18,19]
	Providing information on attractions & shopping in the city (E2)	Providing information on places that can be visited during non-match times, allowing participants to flexibly arrange their free time.	[18,19]
	Increasing employment opportunities for urban residents (E4)	Local residents serve as staff during sports events, increasing employment opportunities for local residents.	[8,9,18,24]
	Sponsorship and support from local businesses (E5)	Local companies support the development of urban sports and provide more event sponsorship, funding and assistance.	[3]
	Sponsored Brand Exposure (E6)	Logos of sponsoring companies are placed in or around the venue, or sports merchandises are provided by the brands.	[3]
	Increasing the number of visits to the attractions in the city (E7)	Enhancing the richness of attractions around the city to attract more people and increase visits.	[9,24]
Institutional (I)	Combined with smart wearable device (I1)	Smart devices are used in sports events to monitor the physiological status and conditions of the contestants.	Experts’ opinions
	Maintenance of urban tourism website (I2)	Maintaining and updating information on urban sports events.	[3,19]
	Enhancing participant reward system (I3)	Increasing the prizes and bonuses of the event to increase participants’ willingness to participate.	[18,19]
	In conjunction with festivals in the city (I4)	Urban sports events combined with local festivals and events can bring participants richer experiences.	[9,18,24]
	Promotion of urban culture and heritage (I5)	Developing plans for the promotion of the city’s historical culture and heritage.	[8,9,24]
	Land planning for sports events (I6)	Drawing up complete protection measures for the event venue and clearly marking the event areas and related events.	[8,24]
	Marketing and promotion by local governments (I7)	Local governments organize sporting events from time to time and plan marketing strategies.	[3,24]

Source: authors’ own compilation.

**Table 2:** BWM evaluation ratings.

Linguistic Variable	Crisp Value
Equally important	1
Equal to moderately more important	2
Moderately more important	3
Moderately to strongly more important	4
Strongly more important	5
Strongly to very strongly more important	6
Very strongly more important	7
Very strongly to extremely more important	8
Extremely more important	9

**Table 3:** DEMATEL’s evaluation ratings.

Linguistic Variable	Crisp Value
No influence	0
Low influence	1
Medium influence	2
High influence	3
Very high influence	4

**Table 4:** Criteria weights obtained through Bayesian BWM.

Dimension	Criteria (Weight)	Ranking	Dimension	Criteria (Weight)	Ranking
**Social (S)**	S1 (0.086)	7	**Environmental (G)**	G1 (0.129) *	4 *
	S2 (0.087)	6		G2 (0.137) *	3 *
	S3 (0.090)	5		G3 (0.070)	8
	S4 (0.116)	4		G4 (0.182) *	2 *
	S5 (0.054)	8		G5 (0.075)	7
	S6 (0.143) *	3 *		G6 (0.203) *	1 *
	S7 (0.202) *	2 *		G7 (0.078)	6
	S8 (0.223) *	1 *		G8 (0.126) *	5 *
	α-cut = 0.125			α-cut = 0.125	
**Economic (E)**	E1 (0.096)	5	**Institutional (I)**	I1 (0.194) *	3 *
	E2 (0.090)	6		I2 (0.150) *	4 *
	E3 (0.083)	7		I3 (0.071)	7
	E4 (0.196) *	2 *		I4 (0.204) *	2 *
	E5 (0.204) *	1 *		I5 (0.086)	5
	E6 (0.165) *	4*		I6 (0.074)	6
	E7 (0.167) *	3 *		I7 (0.220) *	1 *
	α-cut = 0.143			α-cut = 0.143	

Note: The “*” symbol represents the criteria that exceed the threshold value. These criteria would be calculated by DEMATEL.

**Table 5:** Criterion screening results for three different methods.

Method	(Criteria Through Screening)
AHP	S6, S7, S8, G1, G2, G4, G6, E4, E5, E6, E7, I1, I4 and I7
BWM	S6, S7, S8, G1, G2, G4, G6, E4, E5, E6, E7, I1, I4 and I7
Bayesian BWM (This study)	S6, S7, S8, G1, G2, G4, G6, G8, E4, E5, E6, E7, I1, I2, I4 and I7

**Table 6:** Sum of the defuzzification of rough influences given and received by criteria.

	${\tilde{s}}_{i}$	${\tilde{o}}_{i}$	${\tilde{s}}_{i}+{\tilde{o}}_{i}$	${\tilde{s}}_{i}-{\tilde{o}}_{i}$	*s_i_*	*o_i_*	*s_i_* + *o_i_*	*s_i_* − *o_i_*
S6	[0.581, 2.551]	[0.759, 2.539]	[1.340, 5.090]	[−1.958, 1.792]	1.566	1.649	3.215	−0.083
S7	[0.424, 2.055]	[0.828, 2.848]	[1.252, 4.903]	[−2.423, 1.228]	1.240	1.838	3.078	−0.598
S8	[0.788, 3.000]	[0.895, 3.116]	[1.683, 6.116]	[−2.328, 2.105]	1.894	2.006	3.900	−0.112
G1	[0.866, 3.011]	[0.755, 2.873]	[1.621, 5.884]	[−2.007, 2.256]	1.939	1.814	3.753	0.125
G2	[0.790, 2.761]	[0.891, 2.850]	[1.681, 5.611]	[−2.059, 1.870]	1.776	1.870	3.646	−0.095
G4	[0.466, 2.112]	[0.643, 2.413]	[1.109, 4.525]	[−1.947, 1.468]	1.289	1.528	2.817	−0.240
G6	[0.740, 2.935]	[1.129, 3.476]	[1.869, 6.411]	[−2.736, 1.806]	1.837	2.303	4.140	−0.465
G8	[0.467, 2.121]	[0.645, 2.683]	[1.112, 4.804]	[−2.216, 1.476]	1.294	1.664	2.958	−0.370
E4	[0.643, 2.944]	[0.883, 2.987]	[1.526, 5.931]	[−2.344, 2.061]	1.793	1.935	3.728	−0.142
E5	[1.049, 3.646]	[0.938, 3.140]	[1.986, 6.786]	[−2.091, 2.708]	2.347	2.039	4.386	0.308
E6	[0.813, 2.604]	[0.815, 2.843]	[1.629, 5.447]	[−2.030, 1.789]	1.709	1.829	3.538	−0.120
E7	[1.423, 3.935]	[1.068, 3.389]	[2.491, 7.323]	[−1.966, 2.867]	2.679	2.228	4.907	0.450
I1	[0.389, 1.832]	[0.236, 1.789]	[0.624, 3.621]	[−1.401, 1.596]	1.110	1.012	2.122	0.098
I2	[1.200, 3.683]	[1.056, 3.392]	[2.256, 7.075]	[−2.192, 2.628]	2.442	2.224	4.665	0.218
I4	[1.501, 3.910]	[0.900, 2.949]	[2.401, 6.860]	[−1.448, 3.010]	2.706	1.925	4.631	0.781
I7	[1.499, 3.820]	[1.200, 3.632]	[2.699, 7.453]	[−2.133, 2.621]	2.660	2.416	5.076	0.244
